# BLAST-QC: automated analysis of BLAST results

**DOI:** 10.1186/s40793-020-00361-y

**Published:** 2020-08-12

**Authors:** Behzad Torkian, Spencer Hann, Eva Preisner, R. Sean Norman

**Affiliations:** Department of Environmental Health Sciences, University of South Carolina, 921 Assembly Street, Columbia, SC 29208 USA

**Keywords:** Basic Local Alignment Search Tool, Blast, Bioinformatics, Genomics, Sequencing

## Abstract

**Background:**

The Basic Local Alignment Search Tool (BLAST) from NCBI is the preferred utility for sequence alignment and identification for bioinformatics and genomics research. Among researchers using NCBI’s BLAST software, it is well known that analyzing the results of a large BLAST search can be tedious and time-consuming. Furthermore, with the recent discussions over the effects of parameters such as ‘-max_target_seqs’ on the BLAST heuristic search process, the use of these search options are questionable. This leaves using a stand-alone parser as one of the only options of condensing these large datasets, and with few available for download online, the task is left to the researcher to create a specialized piece of software anytime they need to analyze BLAST results. The need for a streamlined and fast script that solves these issues and can be easily implemented into a variety of bioinformatics and genomics workflows was the initial motivation for developing this software.

**Results:**

In this study, we demonstrate the effectiveness of BLAST-QC for analysis of BLAST results and its desirability over the other available options. Applying genetic sequence data from our bioinformatic workflows, we establish BLAST_QC’s superior runtime when compared to existing parsers developed with commonly used BioPerl and BioPython modules, as well as C and Java implementations of the BLAST_QC program. We discuss the ‘max_target_seqs’ parameter, the usage of and controversy around the use of the parameter, and offer a solution by demonstrating the ability of our software to provide the functionality this parameter was assumed to produce, as well as a variety of other parsing options. Executions of the script on example datasets are given, demonstrating the implemented functionality and providing test-cases of the program. BLAST-QC is designed to be integrated into existing software, and we establish its effectiveness as a module of workflows or other processes.

**Conclusions:**

BLAST-QC provides the community with a simple, lightweight and portable Python script that allows for easy quality control of BLAST results while avoiding the drawbacks of other options. This includes the uncertain results of applying the -max_target_seqs parameter or relying on the cumbersome dependencies of other options like BioPerl, Java, etc. which add complexity and run time when running large data sets of sequences. BLAST-QC is ideal for use in high-throughput workflows and pipelines common in bioinformatic and genomic research, and the script has been designed for portability and easy integration into whatever type of processes the user may be running.

## Background

The Basic Local Alignment Search Tool (BLAST) from NCBI has been a popular tool for analyzing the large data sets of genetic sequences that have become common when working with new generation sequencing technologies. BLAST has been the preferred utility for sequence alignment and identification in bioinformatics and genomics research and workflows for almost 30 years [1]. One of the main challenges for researchers utilizing the NCBI BLAST is interpreting the huge amount of output data produced when analyzing large numbers of input sequences. While BLAST does allow for multiple output formats as well as limiting the number of top hit results (using -outfmt and -max_target_seqs, respectively) [2], for some purposes such as pushing results down a workflow or pipeline, these tools may not be enough to ensure results that can be meaningfully and reasonably interpreted. The controversy raised by Shah et al. [3] in their 2018 paper outlining a bug in the functionality of the -max_target_seqs parameter has started a discussion in the BLAST community over the usage and potential for misuse of the parameter. NCBI published a response stating that the utility of this parameter is simply misunderstood by the community and that the bug seen by Shah et al. was the result of “overly aggressive optimization” introduced in 2012, and patched the issue following the release of BLAST+ 2.8.1 in 2018 [4]. However, follow up test cases and posts, including those by Peter Cock [5], have shown that this issue is much more complex than simply “BLAST returns the first N hits that exceed the specified e-value threshold”. While the update 2.8.1 fixed 9/10 of Shah et al’s test cases, according to the post by Peter Cock, 1/10 remained invalid, due to an error with the internal candidate sequence limit introduced by -max_target_seqs 1. This is because, as was stated by Shah et al. [3], the -max_target_seqs parameter is applied much earlier in the search, before the final gapped alignment stage. This means that the use of this parameter can change the number of sequences processed as well as the statistical significance of a hit if using composition-based statistics [2]. This is contrary to the popular assumption that the parameter is simply a filter applied post search [6]. This intuition is false, and may lead to errors in the resulting data of a BLAST search if the value of -max_target_seqs is too small. The use of -max_target_seqs in this way is not advised. As a result of the misinformation and confusion over ‘-max_target_seqs’ and other intricacies of the BLAST heuristic search and filtering process, there has been a push towards more detailed documentation of these processes and effects of parameters on the BLAST algorithm [6], with NCBI adding a warning to the BLAST command-line application if the value of -max_target_seqs is less than 5 [7]. The community has also moved towards better methods of narrowing the results of a large search, as opposed to using BLAST parameters that may affect the actual search process. These methods include resources like Bio-Perl and BioPython that can be used to create scripts to parse and filter result files. A few community written scripts can be found available online, such as the Perl script created by Dr. Xiaodong Bai and published online by Ohio State [8], a version of this script produced by Erin Fichot [9], and a XML to tabular parser by Peter Cock [10]. While all of these scripts (and others like them) can potentially be very useful for parsing BLAST XML results into a concise tabular format, most have drawbacks that leave much to be desired. First and most importantly, for Bai and Fichot, the programs require Perl and Bio-Perl modules which can be unwieldy and slow for use in high-throughput workflows and pipelines, especially those which are built on a modern python framework. Furthermore, both scripts contain a bug, found on lines 77 and 93 respectively, that causes the query frame value to be lost through the parsing process, setting the value to 0. Our team sought to correct this and other errors and to provide a verified solution that can be soundly applied for research purposes. Secondly, the team saw a need for increased functionality, particularly the ability to filter results by threshold values input by the user. The only program that implements a threshold other than BLAST-QC is the script by Fichot, but only a bit score threshold is implemented. Our team sought to provide a single solution that would let researchers determine the best combination of values that would be optimal for any given experiment without the need to change parsers between runs. The team’s central motivation was to pursue creating a dedicated piece of quality control software for use in research workflows, find a solution that solely utilizes Python 3, streamlines the process, and reduces run times for parsing large data sets of BLAST results. Implementation:

BLASTQC has been implemented in a single python file, requiring only that Python3 be installed for all functionalities to be used. The team felt that an implementation in Python was important for the simplicity and ease of use that comes with the Python environment. Python is also one of the most popular and well understood languages for research purposes, and thus is a perfect choice for a tool that is designed for portability and integration into other research processes. Python is also capable of very fast runtimes when compared to other interpreted languages, such as Perl, and while it may be slower than a compiled language like C, the benefits in ease of use and portability outweigh the minor increase in runtimes. For example, C requires the use of dependencies like libxml2 for parsing, requiring a higher level of knowledge to make modifications to source code, and as such is less desirable as a simple addition to bioinformatic workflows already built within the Python framework. With Python 3 the parsing step of the workflow is simplified to a single file. Furthermore, the use of a standalone script rather than the use of a command line sorting option such as GNU sort not only provides a great increase in possible functionality, as implementing filtering parameters in bash on the command line can be cumbersome, but also allows for a better user experience for researchers who don’t want to memorize long sort commands that need to be changed constantly as experiment goals change. The BLAST-QC script implements thresholds on e-value, bit-score, percentage identity, and the number of ‘taxids’ (level of taxonomic or functional gene detail) in the definition of a hit (<hit_def > in BLAST XML results). It is also possible for the user to choose which of these values the output should be ordered by and how many top matches should be returned per query sequence in the input. Thus, the behavior of the -max_target_seqs parameter may be implemented with ease without altering the search process. Additionally, if the researcher decides that a higher bit-score is more important for a certain experiment, it is trivial to change the parsing process to return the highest bit-score hit, whereas max_target_seqs only supports returning top hits by e-value. Further, the Python script is also capable of setting a range on the threshold values, and selecting those sequences that produced a more detailed hit definition within that range. This is useful for researchers because it avoids the problem of finding a high scoring sequence that provides no relevant information, as there may be little use in knowing that a hit accurately matches an unknown sequence. For example, a BLAST search may return a hit sequence with a taxid of “protein of unknown function DUF1680”. This may not be a useful result for a study on the function of a specific protein, regardless of how low the evalue of the hit. BlastQC allows researchers to define the reasonable evalue for their application using input parameters, and returns hits with more informative taxids that still fit within the chosen parameters. Increased definition information is useful for narrowing the taxonomy of a species (for BLAST nucleotide) or the type/functionality of a protein sequence in a protein BLAST query. The team also found an issue in many of the available community parsers regarding the number of HSPs (high scoring pairs) per hit. In some cases BLAST may return multiple HSPs per hit sequence, and the BLAST-QC script handles this by considering it a separate hit that retains the same id and definition. This case was not covered in any of the scripts the team encountered online, causing hits with multiple HSPs to lose any data from the additional HSPs. The BLAST-QC Python script is compatible with all BLAST types (BLASTP, BLASTN, BLASTX, etc.) as well as both the tabular and XML output formats (−outfmt 6 and -outfmt 5, respectively) and reports all relevant data produced in a BLAST results file: query name, query length, accession number, subject length, subject description, e-value, bit-score, query frame, query start, query end, hit start, hit end, percent identity, and percent conserved (qseqid, sseqid, pident, length, mismatch, gapopen, qstart, qend, sstart, send, evalue, bitscore and salltitles (optional) for tabular output). Information on these values can be found in the BLAST glossary and manual [2, 11], and the two percentage values (percent identity and percent conserved) have been calculated using the identity (Hsp_identity), positive (Hsp_positive) and align length values (Hsp_align-len). Percent identity is defined as the percent of the sequences that have identical residues at the same alignment positions and is calculated by the number of identical residues divided by the length of the alignment multiplied by 100 (100*(hsp_identity/hsp_align-len)). Percent conserved (positive) is defined as the percent of the sequences that have ‘positive’ residues (chemically similar) at the same alignment positions and is calculated by the number of positive residues divided by the length of the alignment multiplied by 100 (100*(hsp_positive/hsp_align-len)). Additionally, BLAST-QC supports parallel processing of results, using Python’s multiprocessing module. The number of concurrent BLASTQC processes defaults to the number of CPU cores present on the machine, but this value may be adjusted using the -p command line option. If sequential processing is desired, the number of processes may be set to 1 using “-p 1”. This along with the ability to pipe input from stdin allow for replication of some of GNU sort’s main features.

## Results

The objective of the development of BLAST-QC was to provide BLAST users with a method of quality control that ensures accuracy of results, posts superior runtimes, and provides configurations for many types of analysis processes, while remaining streamlined and simple to use and modify. In order to establish BLAST-QCs effectiveness as compared to other quality control options, we have compared BLAST-QC python to implementations of the program in compiled languages, both C and Java, to the community available parsers by Bai, Fichot and Cock, and to a standard approach to parsing for some researchers, GNU sort commands.

**Fig. 1:**
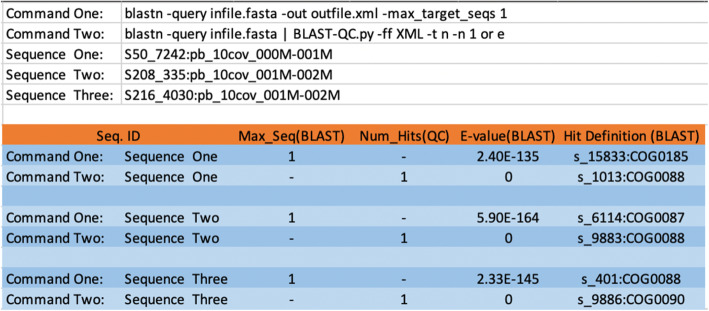
Demonstration of max_target_seqs error and correction (BLAST 2.8.0 or earlier) on the test cases presented by Shah et al. in 2018 [3]. Command 1 demonstrates the usage of BLASTN to produce only 1 top e-value hit per query sequence, using ‘-max_target_seqs 1’. Command 2 demonstrates the usage of BLAST-QC to parse the top e-value hit from the standard BLAST output, using ‘-or e -n 1’ to order by e-value and return only 1 top hit per query. These commands are executed for 3 separate query sequences (represented in the respective commands by infile.fasta), sequences 1, 2 and 3. As the e-values in figure show (column 5), BLAST-QC is able to extract the top e-value hit from these test case examples, while max_target_seqs is not. This error has been corrected in BLAST 2.8.1, but we show this example for compatibility with those who might be running older versions of BLAST or have older BLAST results datasets

We demonstrate the ability of BLAST-QC to correct the issue with `max_target _seqs`, using the dataset provided in the case study from Shah et al. [3]. This dataset is available on Shah’s github page, ‘https://github.com/shahnidhi/BLAST_maxtargetseq_analysis’. As shown in Fig. 1, BLAST-QC was able to correctly identify the lowest e-value hit for the same query sequence while BLAST with ‘-max_target_seqs = 1’ was not. This result illustrates the potential for errors to be introduced into BLAST data by the use of this parameter, and we encourage researchers to seek more information on its usage and application [6, 11]. To use BLAST-QC to replicate the function of ‘-max_target_seqs’ leave the parameter set to default while using BLAST to locate matching sequences, then run BLAST-QC on the resulting data, ordering output by e-value and setting a limit of 1 hit per query using the syntax shown at the bottom of Fig. 1. Although the issue with max_target_seqs has been corrected in BLAST 2.8.1, it is still not well understood by the BLAST community and, being a popular parameter, we felt that it was important to show this use case in order to demonstrate a safe way of achieving the desired effect, as well as to promote compatibility with older versions of the BLAST software. We also demonstrate the runtime of BLAST-QC as compared to existing parsers, two developed with the commonly used BioPerl modules, one written by Xiaodong Bai from Ohio State and an improved version of the same script developed by Erin Fichot [9], an XML to tabular conversion script published by Peter Cock [10], implementations of the BLAST-QC parser in both Java and C, as well as a standard GNU sort command. The implementations in C and Java were necessary to compare BLAST-QC Python to both compiled and interpreted languages, as both Python and Perl are interpreted languages. The sort command used for the runtime benchmarking is “sort -k1,1 -k11,11 blast.tab”, as this replicates ordering the hit sequences for each query sequence by evalue, BLAST-QCs default mode. Thus, we sort by query name first, then by evalue. All runtime data was gathered using a system with a 28 core Intel Xeon E5–2680 @ 2.4GHz and 128GB of RAM. All sample datasets used for figures were produced using nucleotide sequences extracted for use in another one of our teams bioinformatic workflows [9]. The BLAST command used to produce the result data was: ‘ncbi-blast-2.10.0+/bin/blastn -query Aug2013_metaG_12142016-34108099_Experiment1_layer1.fasta -db SILVA_132_SSURef_Nr99_tax_silva_trunc -outfmt (5 and 6) -num_threads 28’. Result datasets were then split into 5 files containing 10^3^, 10^4^, 10^5^, 10^6^ and 10^7^ query sequences respectively. As the datasets used for the runtime tests are very large (the largest being ~60gb for 10^7^ query sequences), we have hosted the datasets on our team’s HPC server. For access to the exact data used for all test cases, please submit a request at: https://sc.edu/about/offices_and_divisions/division_of_information_technology/rci/.

**Fig. 2:**
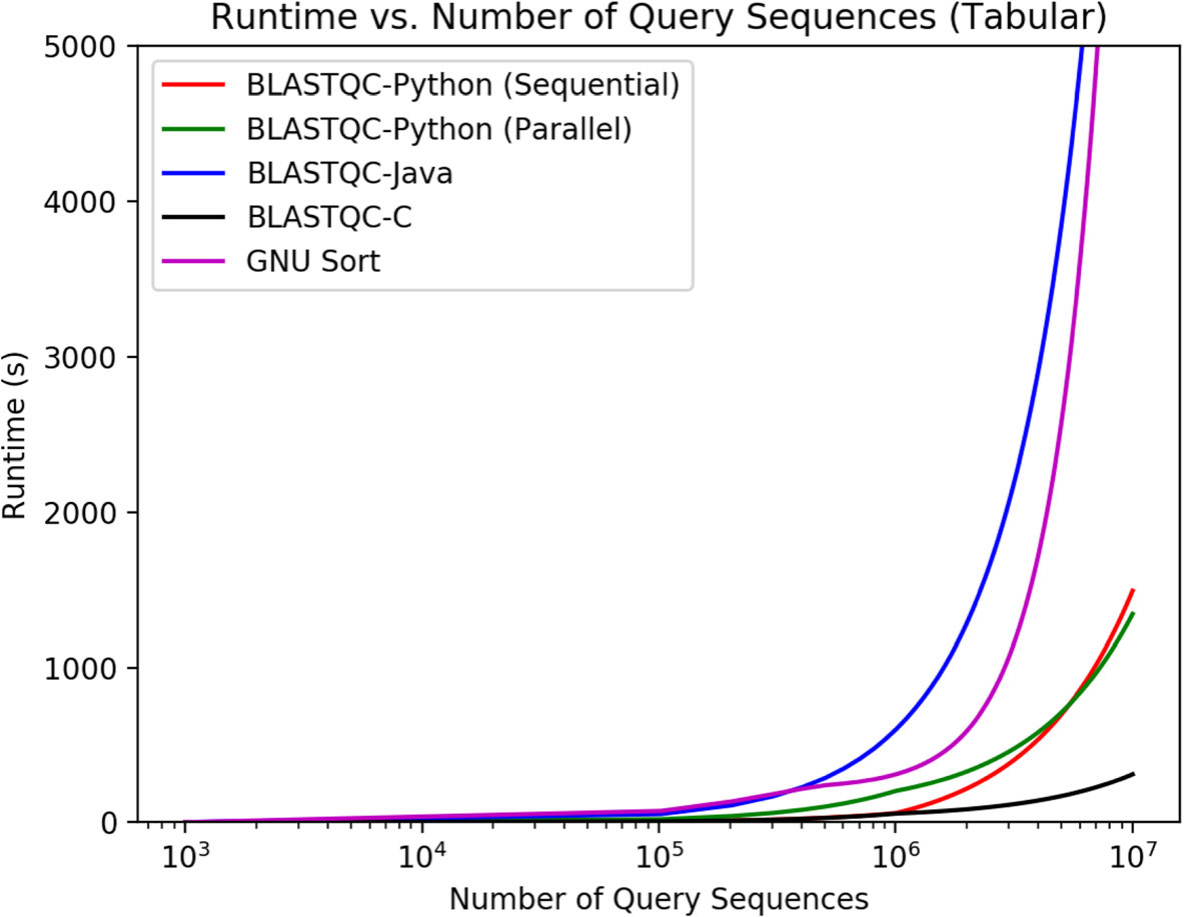
Plot of runtime vs number of query sequences in a BLASTN tabular (−outfmt 6) results file. The graph is a linear-log plot, the number of query sequences is shown on a log scale, due to the exponential nature of runtime data and the large numbers of sequences involved. Each of the scripts were run against BLAST tabular files containing 10^3^, 10^4^, 10^5^, 10^6^ and 10^7^ query sequences, respectively. All versions of BLAST QC were run using default parameters (no command line options specified), which is to order hit sequences for each query by e-value. To replicate this behavior using GNU sort, the command ‘sort -k1,1 -k11,11’, was used as this orders the rows in the tabular output by query id then e-value (the 1st and 11th columns respectively)

**Fig. 3:**
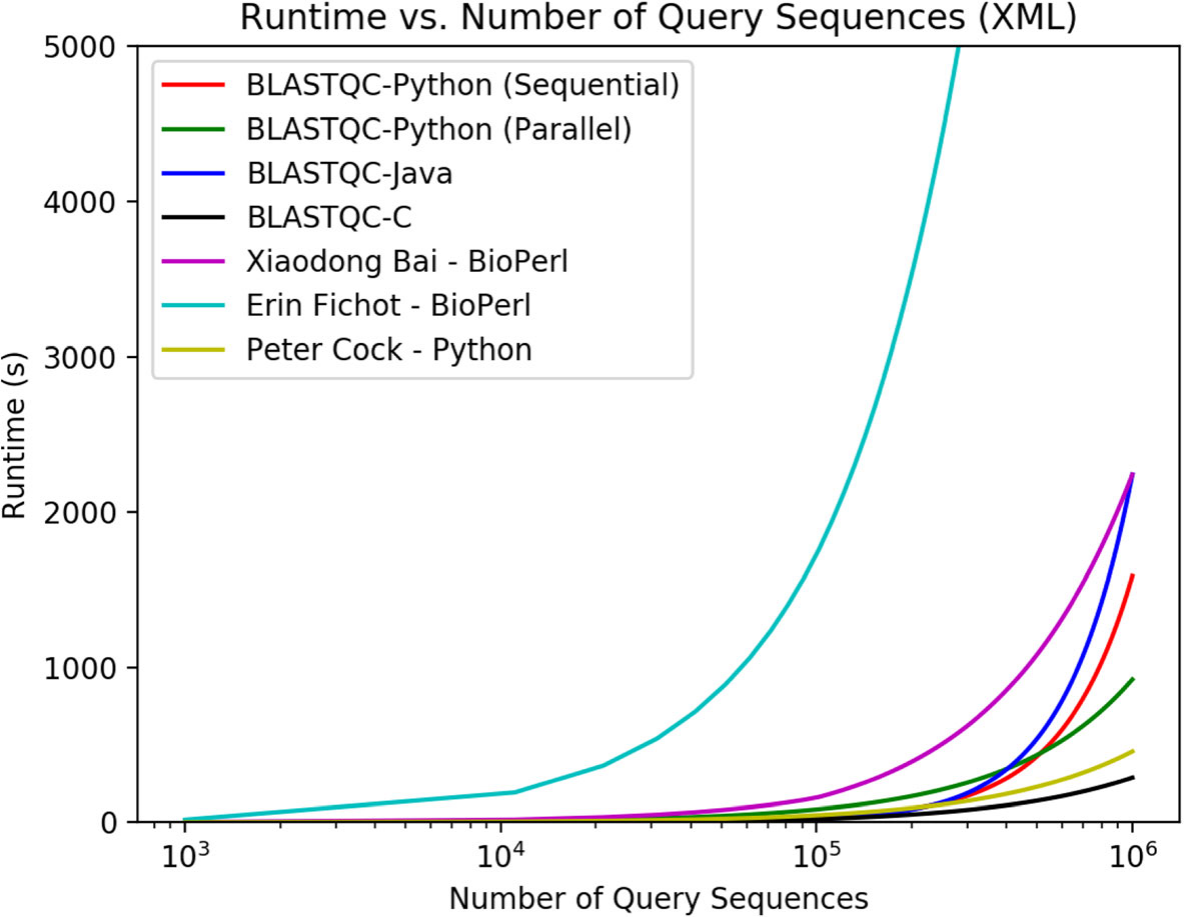
Plot of runtime vs number of query sequences in a BLASTN XML (−outfmt 5) results file. The graph is a linear-log plot, the number of query sequences is shown on a log scale, due to the exponential nature of runtime data and the large numbers of sequences involved. Each of the scripts were run against BLAST XML files containing 10^3^, 10^4^, 10^5^, 10^6^ query sequences respectively. We did not include a run of 10^7^ XML query sequences as the file size became impractical for our system, taking up all 128Gb of ram (this resulted in a outOfMemoryException on the Java parser). All versions of BLAST QC were run using default parameters (no command line options specified), which is to order hit sequences for each query by e-value. Fichot’s BioPerl script was also run using default parameters with no thresholds implemented. Both the BioPerl script by Xiaodong Bai and the Python script by Peter Cock only function as XML (outfmt 5) to tabular format (outfmt 6) converters, so no input parameters are required

**Fig. 4:**
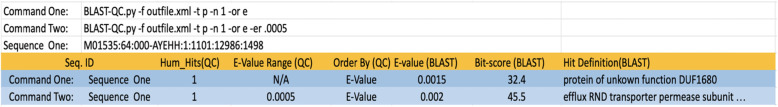
Demonstration of range parameters of BLAST-QC. Command 1 depicts the usage of BLAST-QC to return a single top hit per query sequence. Command 2 depicts the same command with an additional range parameter, ‘-er .0005’. This will return a hit within this e-value range (+.0005 from the top e-value hit) that has the most taxids present. Thus, as the figure shows, the result of command 2 is a hit with a e-value that is .0005 more than the first top hit returned in command 1, but with more informative taxids

**Fig. 5:**
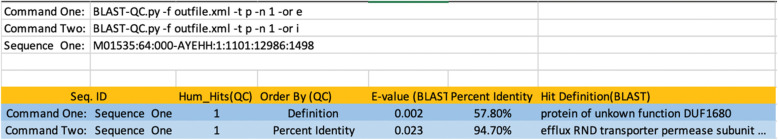
Demonstration of the order by function of BLAST-QC. Command 1 depicts the usage of BLAST-QC to order a BLAST results file by e-value, while command 2 depicts the usage of BLAST-QC to order results by percent identity. The result of each respective command is shown in the table above

**Fig. 6:**
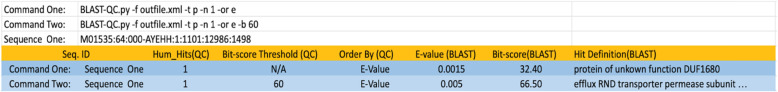
Demonstration of threshold functionality of BLAST-QC. Command 1 demonstrates the usage of BLAST-QC to return the top e-value hit per query, while command 2 depicts the usage of BLAST-QC to return the top e-value hit per query with a bitscore threshold of 60 (−b 60). The results of command 1 return a hit with an e-value of .0015, while the hit’s bitscore is only 32.40. the results of command 2 return a hit with an e-value of .005, but with a bitscore of 66.5

While each script is designed to operate on a BLAST output file, they all differ in functionality and implementation. All versions of BLAST-QC (Python, Java, C) can operate on both XML and tabular BLAST outformats, while the scripts by Bai, Fichot and Cock only operate on XML output, whereas GNU sort only functions on a tabular outformat. These scripts were chosen for comparison as they replicate many of the possible use cases for BLAST-QC, both direct tabular conversion of results as well as the application of filtering thresholds to provide quality control. Both scripts by Bai and Cock do not provide any quality control or threshold functionality, they simply function as XML to tabular format converters for BLAST results, while Fichot implements a bit-score threshold and support for both protein and nucleotide databases. All versions of BLAST-QC implement the ability to operate on both BLAST output formats, the ability to input various filters to narrow results to the highest quality sequences, and support for both protein and nucleotide databases. Figure 2 plots runtime vs number of query sequences for all four programs that operate on tabular format, using the same dataset of BLAST result files. As the figure depicts, BLAST-QC C version is the fastest program, followed by Python, GNU sort then Java. While the C version is certainly faster, it requires the use of external libraries libxml2, requires more knowledge than python to operate and maintain, and is only marginally faster. Most notably, BLASTQC Python outperforms a GNU sort of the dataset at ordering hits by evalue. Many researchers choose GNU sort as a standard approach to parsing BLAST results as it is a widely available solution, but as the figure shows, it does not perform as well as the standalone parser. Furthermore, more complex QC tasks require a strong knowledge of GNU sort’s syntax and creates additional runtime, making standalone parsers much more functional for complex parsing tasks (eg. replicating -max_target_seqs). Lastly, the Java parser performed the worst of the four parsers in the tabular benchmark, despite the fact that Java is a compiled, rather than interpreted, language. This is most likely due to the high memory overhead required by the Java Virtual Machine (JVM), which takes up memory bandwidth that is needed for parsing the large BLAST files. Figure 3 plots runtime vs number of query sequences for all 6 programs that operate on XML format, using the same dataset of BLAST result files. Both Bioperl parsers performed worst out of all the XML parsers, with Fichot’s script being somewhat of an outlier in the dataset (over 4 h to parse a file with 10^6^ query sequences). This is most likely due to the combination of the cumbersome BioPerl modules and the more involved parsing of Fichot’s script as compared to the script by Xaiodong Bai, as well as the fact that Perl is an interpreted language. The C parser had the fastest runtime in the XML benchmark, followed by the blastxml_to_tabular. The blastxml_to_tabular script simply converts the data from XML to tabular, so no real computations are required. In both Figs. 2 and 3, we plot runtimes for both sequential (1 core) and parallel processing modes (28 cores) of the BLAST-QC python application, to demonstrate the effect of the parameter on runtime. While the parallel processing takes more time for a lower number of query sequences, at a value of approximately 5.055 × 10^5^ query sequences for XML out format, and 5.354 × 10^6^ for tabular, the program running in parallel achieves a faster runtime than that of the sequential program. This is due to the fact that opening each separate process and facilitating communication creates more overhead than the parallelization can scale for smaller inputs, but over a larger number of query sequences the efficiency of parallelization decreases the overall runtime, despite the overhead of the required process management. In Figs. 4-6 we demonstrate some of the various functionalities of BLAST-QC and provide the results of their application to a sample dataset. In Fig. 4 the range functionality is demonstrated using an e-value range of .0005. This means that BLAST-QC will consider hits found that fall within that range of the lowest e-value hit, if the target sequence provides an increase in the quality of the hit definition (taxids in <Hit_def > or salltitles). As depicted in Fig. 5 the sequence returned by simply returning the lowest e-value hit provides a definition that may not be useful for research analysis, while the top hit using a range value provides an insightful description of the sequence while maintaining a reasonable e-value within that range. In BLAST-QC, range values are implemented on e-value, bit-score, and percent identity. Figure 5 depicts the ordering functionality of BLAST-QC, and ordering by e-value, bit-score, percent identity and hit definition is implemented. For example, when ordering by definition, as in Fig. 5 [1], the hit that has the highest quality of hit definition that fits input thresholds will be returned. Figure 6 demonstrates the threshold capability of the BLAST-QC program. The first sequence is returned when ordering by e-value with the number of hits set to one (replicating max_target_seqs). The second employs a bit-score threshold to find a matching sequence with the highest e-value that also has a bit-score above the threshold. Threshold values are implemented on e-value, bit-score, percent identity and hit definition. All of the resources needed for the BLAST-QC software are available for download from the BLAST-QC GitHub repository: https://github.com/torkian/blast-QC. Additional test-cases and usage information for the program are located on the page as well.

## Discussion

BLAST-QC was developed using Python 3, and is designed for usage within a script or larger workflow, but also offers a headless command-line interface for use with smaller datasets. Usage information has been documented in this paper, and additional documentation, as well as all test-cases and datasets used in this paper can be found in the BLAST-QC GitHub repository, at https://github.com/torkian/blast-QC. Our team seeks to standardize researchers’ approach to analyzing BLAST result datasets. Many researchers opt to apply ‘max_target_seqs’ as a quality control parameter in their research workflows (over 400 Google Scholar papers reference the value) [12–14], even though it has been shown that this parameter can cause issues with the search process and resulting data, and is simply not intended for this purpose. Those who use a standalone script are exchanging gains in the accuracy of results and greater functionality and control over parameters for increased bulk and runtime, which add up when running large datasets of sequences. With the added functionality and superior runtime that BLAST-QC provides over an option like GNU sort or max_target_seqs, the BLAST-QC script provides a practical option for parsing of BLAST result files, especially as with the use of Python, the task can be simplified to a single file. While there are other standalone quality control and filtering options available for BLAST results, BLAST-QC python takes a novel approach to the task, eliminating the necessity for other dependencies and allowing researchers to have increased control over the level of definition of results, while also providing greatly decreased runtimes when compared to other language and parsing options. We encourage the community to consider available options when seeking analysis of BLAST results, and to help contribute to and improve on our source code by submitting a pull request on the BLAST-QC GitHub page.

## Conclusions

BLAST-QC provides a fast and efficient method of quality control for NCBI BLAST result datasets. It offers greater functionality for controlling the desired QC parameters when compared to existing options and outperforms them in terms of runtime. We suggest that it is BLAST-QC’s Python 3 framework that allows it to outperform dense BioPerl and BioPython modules, while it also provides much higher functionality than GNU sort or even -max_target_seqs. Furthermore, BLAST-QC provides seamless integration into larger workflows developed with Python 3. With the increase in popularity of high-performance computing and new generation sequencing, novel approaches to BLAST quality control and other bioinformatic computational processes are needed to handle the increasing size of datasets, but also to take advantage of the increasing capacity of computing to provide solutions to these problems. Our team also sought to increase awareness of the controversy surrounding the application of the ‘max_target_seqs’ parameter in BLAST, and to provide a sound solution that replicates the function of the parameter and ensures the highest quality results. The BLAST-QC software and all other documentation and information can be located at BLAST-QC’s GitHub page.

## Data Availability

- Project name: BLAST-QC. - Project home page: https://github.com/torkian/blast-QC - Operating system(s): Platform independent. - Programming language: Python 3. - Other requirements: None. - License: GNU GPL. - Any restrictions to use by non-academics: None.

## Abbreviations

BLAST: Basic Local Alignment Search Tool
QC: Quality Control
XML: eXtensible Markup Language
HSP: High Scoring Pairs
HPC: High-Performance Computing
JVM: Java Virtual Machine

## References

[1] Altschul, Stephen F, et al. “Basic Local Alignment Search Tool.” Journal of Molecular Biology, Academic Press, 6 Feb. 2007, www.sciencedirect.com/science/article/pii/S0022283605803602?via%3Dihub.

[2] BLAST® Command-Line Applications User Manual., National Center for Biotechnology Information, US National Library of Medicine, 14 Nov. 2018, www.ncbi.nlm.nih.gov/books/NBK279684/.

[3] Shah, Nidhi, et al. “Misunderstood Parameter of NCBI BLAST Impacts the Correctness of Bioinformatics Workflows.” OUP Academic, Oxford University Press, 24 Sept. 2018, academic.oup.com/bioinformatics/article/35/9/1613/5106166. 10.1093/bioinformatics/bty833 30247621

[4] Madden, Thomas L, et al. “Reply to the Paper: Misunderstood Parameters of NCBI BLAST Impacts the Correctness of Bioinformatics Workflows.” OUP Academic, Oxford University Press, 24 Dec. 2018, academic.oup.com/bioinformatics/advance-article/doi/10.1093/bioinformatics/bty1026/5259186. 10.1093/bioinformatics/bty1026 PMC6662297 30590429

[5] Cock, Peter. What BLAST’s max-target-sequences Doesn't do, 2015, blastedbio.blogspot.com/2015/12/blast-max-target-sequences-bug.html.

[6] González-Pech RA, Stephens TG, Chan CX (2019). Commonly misunderstood parameters of NCBI BLAST and important considerations for users. Bioinformatics.

[7] Camacho, Christiam. “BLAST+ Release Notes.” BLAST® Help [Internet], U.S. National Library of Medicine, 16 Dec. 2019, www.ncbi.nlm.nih.gov/books/NBK131777/.

[8] Bai, Xiaodong. Ohio State University, Blast_Parsing_for_Modification, https://tomato.cfaes.ohio-state.edu/HCS806/blast_parsing_for_modification_pl.txt. Accessed 16 July 2019.

[9] Preisner, Eva C, et al. “Microbial Mat Compositional and Functional Sensitivity to Environmental Disturbance.” Frontiers in microbiology vol. 7 1632. 17 Oct. 2016, 10.3389/fmicb.2016.01632. 10.3389/fmicb.2016.01632 PMC5066559 27799927

[10] Cock, Peter J. “Peterjc/galaxy_blast.” GitHub, 19 Feb. 2019, github.com/peterjc/galaxy_blast/blob/master/tools/ncbi_blast_plus/blastxml_to_tabular.py.

[11] Fassler, Jan, Peter Cooper. “BLAST Glossary.” BLAST® Help [Internet]., National Center for Biotechnology Information, U.S. National Library of Medicine, 14 July 2011, www.ncbi.nlm.nih.gov/books/NBK62051/.

[12] Meisel JS, Hannigan GD, Tyldsley AS, San Miguel AJ, Hodkinson BP, Zheng Q, Elizabeth A (2016). Grice, Skin Microbiome Surveys Are Strongly Influenced by Experimental Design. J Invest Dermatol.

[13] Long, Kyle A., Nossa, Carlos W., Sewell, Mary A., Putnam, Nicholas H., Ryan, Joseph F., Low coverage sequencing of three echinoderm genomes: the brittle star Ophionereis fasciata, the sea star Patiriella regularis, and the sea cucumber Australostichopus mollis, GigaScience. December 2016;5(1). https://gigascience.biomedcentral.com/articles/10.1186/s13742-016-0125-6. 10.1186/s13742-016-0125-6 PMC4863316 27175279

[14] Giolai, Michael, et al. “Comparative Analysis of Targeted Long Read Sequencing Approaches for Characterization of a Plant’s Immune Receptor Repertoire.” BMC Genomics, BioMed Central, 26 July 2017, 10.1186/s12864-017-3936-7. PMC5530509 28747151

